# The Context-Dependent Regulation and Function of Notch Signaling as a Molecular Mediator of Tumor Plasticity

**DOI:** 10.3390/cells15211945

**Published:** 2026-10-26

**Authors:** Diana Bellavia, Sabrina Zema, Maria Valeria Giuli, Giulia Ferrera, Samantha Cialfi, Saula Checquolo, Rocco Palermo, Claudio Talora

**Affiliations:** 1 Department of Molecular Medicine, Sapienza University of Rome, 00161 Rome, Italy; diana.bellavia@uniroma1.it (D.B.); sabrina.zema@uniroma1.it (S.Z.); giulia.ferrera@uniroma1.it (G.F.); samantha.cialfi@uniroma1.it (S.C.); 2 Department of Medical-Surgical Sciences and Biotechnologies, Sapienza University of Rome, 04100 Latina, Italy; mariavaleria.giuli@uniroma1.it (M.V.G.); saula.checquolo@uniroma1.it (S.C.)

**Keywords:** Notch signaling, tumor plasticity, adaptation

## Abstract

**Highlights:**

**What are the main findings?**

- Notch signaling, rather than acting as a binary regulator, operates as a highly context-dependent system that can be dynamically activated, attenuated, or reconfigured to support distinct cellular responses.
- This dynamic tunability may preserve a broader repertoire of phenotypic responses, allowing Notch to contribute to different adaptive cellular states rather than to a single fixed tumor phenotype.

**What are the implications of the main findings?**

- Tumor evolution arises from stochastic variation, but the trajectory followed by a tumor is shaped by the selective pressures imposed by its environment. Within this process, Notch may provide the phenotypic flexibility that enables tumor cells to adapt to changing selective conditions.
- Notch may therefore represent a paradigmatic model of how dynamically tunable signaling systems contribute to tumor plasticity, raising the possibility that context-dependent modulation of Notch signaling could influence tumor adaptation to therapeutic pressures.

**Abstract:**

In the concept of cancer evolution is implicit a causal necessity that dictates the tumor’s evolutionary trajectory. It is recognized as a dynamic process in which tumor cells continuously adapt to environmental, metabolic, and therapeutic pressures. Such adaptability relies on the progressive engagement of molecular mechanisms that under physiological conditions govern cellular flexibility and fate transitions. During tumorigenesis these regulatory systems are frequently repurposed and integrated into novel functional networks that extend far beyond their canonical roles. From this perspective, the complexity of cancer evolution does not reside merely in the accumulation of genetic alterations, but in the ability of tumor cells to preserve the plasticity necessary to continuously redefine their biological identity. The ability to switch between distinct phenotypic states and respond to selective pressures represents a fundamental hallmark of cancer progression. Among the signaling pathways implicated in these adaptive processes, Notch signaling might play a particularly prominent role. Traditionally regarded as a master regulator of cell fate specification during development, Notch signaling is increasingly emerging as a broader regulator of cellular adaptability. Rather than functioning exclusively as a developmental determinant or alternatively as a classical oncogene or tumor suppressor, accumulating evidence suggests that Notch acts as a context-dependent adaptive network capable of integrating signals generated by genomic instability, DNA damage, metabolic stress, therapeutic exposure, and microenvironmental interactions. In this review, we propose a conceptual framework in which Notch signaling is interpreted as a molecular system governing tumor adaptability. We discuss how post-translational regulators, including PLK1, PIN1, and ubiquitin-dependent mechanisms, shape Notch activity during cellular stress responses. Here, we discuss how tumor cells dynamically regulate Notch signaling and explore how context-dependent Notch signaling contributes to adaptive cellular programs thereby providing a signaling platform through which tumor cells preserve the phenotypic flexibility that underlies adaptation and evolution.

## 1. Introduction

The traditional view of cancer as a disease driven by the progressive accumulation of genetic alterations has profoundly shaped our understanding of tumor initiation and progression. Oncogenic activation and tumor suppressor loss represent fundamental events underlying malignant transformation. However, the evolutionary success of cancer cells cannot be explained solely by their mutational landscape. Recent studies have demonstrated that the biological impact of driver mutations is highly dependent on the cellular context in which they arise, with pre-existing molecular states influencing whether specific genetic alterations are positively selected, negatively selected, or ultimately fixed during tumor evolution [1]. Consequently, cancer progression is increasingly viewed as a dynamic evolutionary process in which malignant cells continuously adapt to changing environmental conditions and selective pressures. Cancer cells exist within highly dynamic ecosystems characterized by fluctuations in nutrient availability, oxygen tension, immune surveillance, stromal interactions, metabolic constraints, biomechanical forces, and therapeutic interventions. Under these conditions, survival depends not only on proliferative capacity but also on the ability to preserve phenotypic flexibility. The capacity to dynamically modify cellular identity, reconfigure transcriptional and metabolic programs, and transition between distinct biological states has therefore emerged as a critical determinant of tumor fitness [1]. Cellular plasticity plays an important role in tumor evolution, enabling malignant cells to survive and exploit newly available ecological niches. In this context, stemness is widely recognized as a major determinant of tumor progression. However, cancer stemness itself represents a dynamic cellular state maintained through continuous interactions between cancer stem cells (CSCs) and their niche rather than a rigid entity [2]. From this perspective, acquisition of stem-like features may constitute one of the adaptive strategies through which tumor cells expand their phenotypic repertoire and increase their fitness under changing environmental and therapeutic conditions. Thus, tumor plasticity is defined as the ability of malignant cells to coordinate diverse adaptive programs that regulate genomic stability, metabolic flexibility, immune interactions, and phenotypic diversification in response to environmental stress. Through this capacity, tumor cells continuously generate biological heterogeneity from which evolutionarily advantageous states can emerge and be selected under changing conditions. The molecular systems that sustain such adaptability are obviously not unique to cancer. Rather, they derive from evolutionarily conserved regulatory networks that normally govern tissue homeostasis, regeneration, and cellular responses to stress. During tumorigenesis, these pathways are frequently co-opted and rewired, allowing transformed cells to exploit physiological mechanisms of flexibility and adaptation for pathological purposes. Consequently, many of the genetic and epigenetic alterations recurrently observed across human cancers can be interpreted as events that enhance the adaptive potential of malignant cells. Among the signaling networks implicated in these processes, Notch signaling occupies a particularly intriguing position [3,4]. Since its discovery [3], Notch has been recognized as a master regulator of cell fate decisions, coordinating lineage specification, stem cell maintenance, differentiation, and tissue organization during embryonic development and adult tissue homeostasis. Consequently, the contribution of Notch to cancer has traditionally been interpreted through its well-established pleiotropic properties, through which Notch signaling is involved in stemness, differentiation, and cell fate commitment. Indeed, aberrant Notch activation has been associated with tumor initiation, disease progression, cancer stem cell maintenance, metastatic dissemination, and therapeutic resistance across a broad spectrum of solid and hematological malignancies [5,6]. However, despite extensive investigation, the role of Notch in cancer remains remarkably complex and highly context-dependent. Depending on the cellular context and disease setting, Notch can exhibit either oncogenic or tumor-suppressive functions, promoting proliferation in some contexts while restraining malignant progression in others [7]. Such apparent contradictions have complicated efforts to define a unified model explaining the contribution of Notch signaling to cancer. Importantly, this functional diversity can emerge even in the same tumor type. Sun and colleagues showed that in HNSCC tumors harboring inactivating NOTCH1 mutations, a subset retains wild-type NOTCH1 and displays activation of downstream Notch effectors, with evidence that Notch signaling can support tumor cell growth [8]. Rather than representing a biological paradox, however, these diverse activities may reflect a more fundamental property of the pathway. An increasing body of evidence suggests that the contribution of Notch to cancer progression extends beyond the regulation of developmental programs and stem cell maintenance. Notch signaling repeatedly emerges in biological contexts characterized by adaptation to stress and environmental perturbations, including responses to DNA damage, therapeutic exposure, metabolic stress, inflammatory signals, and microenvironmental changes. These observations suggest that the biological relevance of Notch might not reside exclusively in the regulation of specific cellular programs, but in its ability to support the adaptive flexibility required for tumor evolution. Consistent with this view, in our studies we have shown that Notch signaling interacts with molecular pathways controlling cellular responses to stress. The functional interplay between NOTCH1 and PLK1 during the DNA damage response revealed that NOTCH1 activity is dynamically regulated under genotoxic stress and contributes to cellular adaptation following DNA damage [9]. Likewise, the identification of the PIN1–NOTCH3 axis in platinum-resistant ovarian cancer demonstrated that NOTCH3 signaling can be selectively exploited under therapeutic pressure to promote survival, metastatic dissemination, and treatment resistance [10]. Similarly, in T-cell acute lymphoblastic leukemia (T-ALL), glucocorticoid receptor activation inhibits NOTCH1 expression, and γ-secretase inhibition can restore glucocorticoid sensitivity in resistant cells [11,12], indicating that NOTCH1 dependency is not uniform across cellular conditions. Additional evidence indicates that post-translational regulators such as PIN1, MAML1, ITCH, and ubiquitin-dependent mechanisms continuously reshape Notch signaling according to cellular context, allowing the pathway to generate distinct biological outputs in response to changing conditions [5,10,13]. Recent observations have further expanded the functional landscape of Notch biology. Beyond its established developmental functions, Notch signaling has been implicated in inflammatory responses, mechanotransduction, and adaptive interactions with the tumor microenvironment [14,15,16,17,18,19]. Together, these findings suggest that Notch integrates a remarkably diverse range of biological inputs extending far beyond developmental cues. Collectively, these observations support a conceptual shift in the interpretation of Notch biology in cancer. Notch may represent one of the molecular systems through which tumor cells preserve the adaptive flexibility required to continuously adjust their behavior in response to environmental, metabolic, inflammatory, mechanical, and therapeutic pressures. Within this framework, stemness itself may represent only one manifestation of a broader adaptive function. The recurrent involvement of Notch in DNA damage responses (DDR), therapy resistance, tumor heterogeneity, metastatic competence, and stress adaptation suggests that its principal contribution to cancer progression may reside in its ability to facilitate phenotypic plasticity and sustain the evolutionary fitness of malignant cells. In this review, we discuss the concept that Notch signaling functions as a molecular regulator of tumor adaptability. We examine how post-translational regulatory mechanisms shape Notch activity under conditions of stress and explore the contribution of Notch signaling to the DDR and stemness-associated programs. We discuss how the involvement of Notch signaling in different molecular pathways and cellular programs, in ways that cannot always be described by a simple binary on–off model, may be viewed as a manifestation of the adaptive flexibility of tumor cells that Notch signaling supports. By integrating these observations within an evolutionary framework, we propose that Notch signaling represents a critical component of the molecular machinery through which cancer cells preserve the plasticity required for long-term adaptation, progression, and survival.

## 2. Crosstalk Between NOTCH1 Signaling, DNA Damage Response and Cell-Cycle Regulation

**Figure 1:**
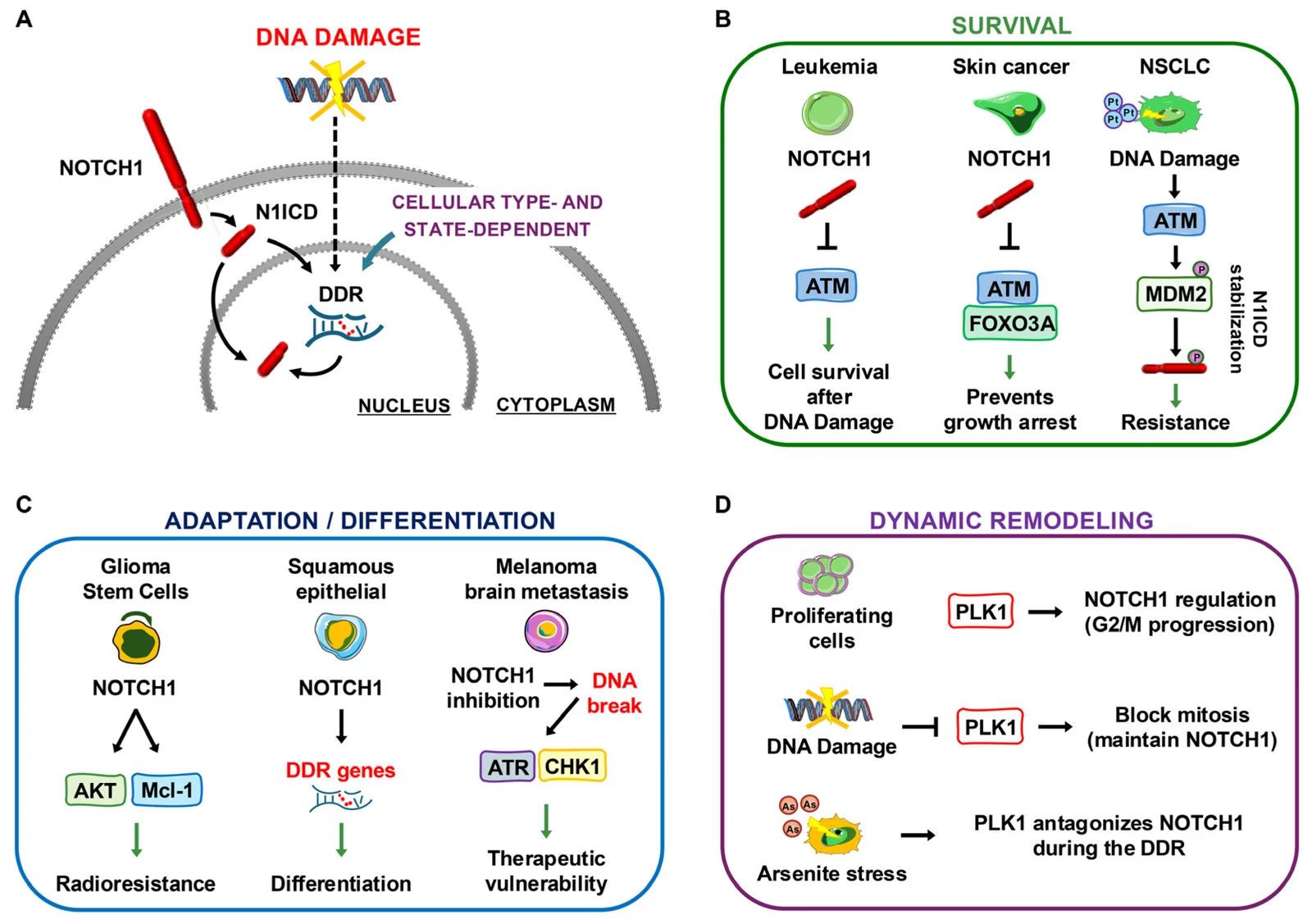
Context-dependent crosstalk between NOTCH1 signaling and DDR. (**A**) NOTCH1 activation releases N1ICD (NOTCH1 intracellular domain), which interacts with the DDR in a cell type- and state-dependent manner. (**B**) NOTCH1 promotes survival following genotoxic stress. NOTCH1 inhibits ATM in T-cell leukemia, thereby supporting cell survival after DNA damage, and it suppresses the ATM–FOXO3A axis in skin cancer-associated fibroblasts, preventing damage-induced growth arrest. In NSCLC, platinum-induced DNA damage activates an ATM–MDM2-dependent mechanism that stabilizes N1ICD and promotes therapeutic resistance. (**C**) NOTCH1 supports adaptive and differentiation-associated programs. It promotes radioresistance through AKT and Mcl-1 in glioma stem cells and it induces DDR-related genes as part of a differentiation program in squamous epithelia. In melanoma brain metastasis, NOTCH1 inhibition induces DNA damage and gives rise to a dependency on ATR–CHK1 signaling, revealing a therapeutic vulnerability. (**D**) PLK1 dynamically remodels NOTCH1 signaling according to cell-cycle and stress conditions. It regulates NOTCH1 during G2/M progression in proliferating cells, while its inhibition upon DNA damage blocks mitotic entry while preserving NOTCH1 expression, and arsenite-induced genotoxic stress triggers a PLK1-dependent mechanism that antagonizes NOTCH1 during the DDR.

The interplay between Notch signaling and the DDR provides an example of how evolutionarily conserved signaling pathways may influence cellular responses to genotoxic stress. One of the earliest indications linking Notch signaling to cellular responses to DNA damage emerged from a genome-wide RNAi screen in *D. melanogaster*, where components of the Notch pathway were identified among the factors required for survival following alkylation-induced DNA damage [20]. These findings suggest that the response to DNA damage cannot be viewed simply as the activation of dedicated repair pathways, but rather as the result of multiple regulatory layers acting in a coordinated manner. Within this framework, evolutionarily conserved signaling systems may be selectively engaged to shape context-dependent adaptive responses, allowing damaged cells to adopt biological programs most compatible with their survival and functional requirements. Further evidence supporting the notion that cellular responses to DNA damage are influenced by developmental signaling pathways emerged from studies on *C. elegans*. In this organism, the GLP-1/Notch pathway plays a central role in maintaining the proliferative germline compartment. Interestingly, following irradiation, proliferative germline cells exhibited an attenuated ATM-dependent response compared with more differentiated meiotic cells [21]. These observations suggest that engagement of DDR pathways is not determined exclusively by the extent of DNA damage itself, but may also depend on cellular state and developmental context. Thus, the biological consequences of genotoxic stress appear to be influenced not only by the nature of the lesion but also by the molecular programs that define cellular identity and proliferative potential. Consistent with this view, Hoffman and colleagues demonstrated that CEP-1, the *C. elegans* orthologue of p53, contributes not only to DNA damage-induced apoptosis but also to efficient DNA repair [22]. Using GLP-1/Notch-driven germline tumors, the authors showed that CEP-1 remains transcriptionally active following genotoxic stress and participates in limiting the accumulation of DNA lesions. Tumors retaining functional CEP-1 displayed reduced lesion burden and decreased tumor size following UVC exposure, whereas loss of CEP-1 resulted in increased genomic instability and enhanced tumor growth. Importantly, these findings indicate that Notch-driven proliferative states can coexist with active genome surveillance mechanisms. Rather than supporting a simple model in which Notch uniformly suppresses DNA damage responses, they suggest that distinct components of the DDR may be differentially engaged according to cellular context and proliferative state [22]. Collectively, these studies suggest that Notch signaling does not simply influence the magnitude of the DNA damage response but may contribute to defining the cellular context in which genotoxic stress is interpreted. Through its ability to regulate proliferative states and cellular identity, Notch signaling may shape the balance between genome maintenance, survival, and adaptation, thereby influencing how cells respond to genomic insults. In mammalian systems, however, the relationship between NOTCH1 and DDR signaling appears to be not so linear (Figure 1A)**.** Several studies indicate that NOTCH1 can attenuate checkpoint activation and thereby favor cellular survival under conditions of genotoxic stress. Vermezovic and colleagues demonstrated that NOTCH1 directly interacts with ATM and negatively regulates its activity independently of canonical transcriptional signaling, promoting survival of Notch-driven leukemic cells following DNA damage [21] (Figure 1B). Similarly, amplification of NOTCH1 in skin cancer-associated fibroblasts suppresses the ATM–FOXO3a axis, preventing damage-induced growth arrest and favoring the expansion of tumor-supportive stromal populations [23] (Figure 1B). Likewise, platinum-induced DNA damage in NSCLC cells stabilizes NICD through an ATM–MDM2-dependent mechanism, linking genotoxic stress to increased NOTCH1 signaling and therapeutic resistance [24] (Figure 1B).

Conversely, other studies suggest that NOTCH1 may also participate in adaptive responses associated with DNA damage signaling. In glioma stem cells, Notch signaling promotes radioresistance through AKT- and MCL1-dependent survival pathways [25] (Figure 1C). In squamous epithelia, NOTCH1 activation induces differentiation-associated programs that include DDR-related genes such as *IER5*, suggesting that DNA damage signaling and differentiation may converge toward coordinated biological outcomes [26](Figure 1C). Moreover, inhibition of NOTCH1 in melanoma brain metastasis induces replication stress and DNA damage, generating a dependency on ATR–CHK1 signaling and revealing a therapeutic vulnerability upon combined pathway inhibition [27] (Figure 1C). Similarly, in BRCA1-deficient triple-negative breast cancer (TNBC), NOTCH1 activation suppresses mitotic catastrophe by restoring S/G2 and G2/M cell-cycle checkpoints through ATR–CHK1 signaling [28] (Figure 1C). Taken together, these observations argue against a linear interpretation of NOTCH1 as either a positive or a negative regulator of DDR signaling. Rather, they suggest that the relationship between NOTCH1 and the DNA damage response is itself highly context-dependent, with distinct cellular states selectively exploiting NOTCH1 to sustain different adaptive outputs. In this framework, the biological consequences of NOTCH1 activation appear to emerge from the integration of genotoxic stress with the broader molecular circuitry that defines cellular identity, proliferative status, and environmental constraints. A particularly illustrative example of this regulatory flexibility emerges from the interplay between NOTCH1 and PLK1 during cell-cycle progression and genotoxic stress. PLK1 acts as a critical regulator of NOTCH1 during both cell-cycle progression and genotoxic stress [9] (Figure 1D). It has been shown that the relationship between NOTCH1 and DDR signaling is not static but dynamically remodeled according to cellular conditions. During unperturbed proliferation, PLK1 contributes to the regulation of NOTCH1 throughout G2/M progression. Following DNA damage, however, inhibition of PLK1 prevents mitotic entry while preserving NOTCH1 expression, thereby uncoupling NOTCH1 activity from proliferative signaling. Remarkably, arsenite-induced genotoxic stress activates a PLK1-dependent mechanism capable of antagonizing NOTCH1 participation in checkpoint signaling, revealing that the functional output of NOTCH1 can itself be actively rewired during the cellular response to DNA damage (Figure 1D). These findings support a model in which NOTCH1 does not impose a predetermined response to genotoxic stress. Rather, its activity appears to be continuously reshaped by the adaptive requirements imposed by the cellular context. From this perspective, NOTCH1 may function as a highly flexible regulatory platform through which cells integrate DNA damage signaling with broader programs controlling checkpoint regulation, survival, differentiation, and proliferative recovery. Consequently, the contribution of NOTCH1 to tumor evolution may not reside in directing a single biological outcome, but in providing malignant cells with an additional regulatory layer through which adaptive responses can be continuously adjusted according to the selective pressures imposed by their environment.

## 3. Notch Signaling and Stress-Induced Cellular Reprogramming

**Figure 2:**
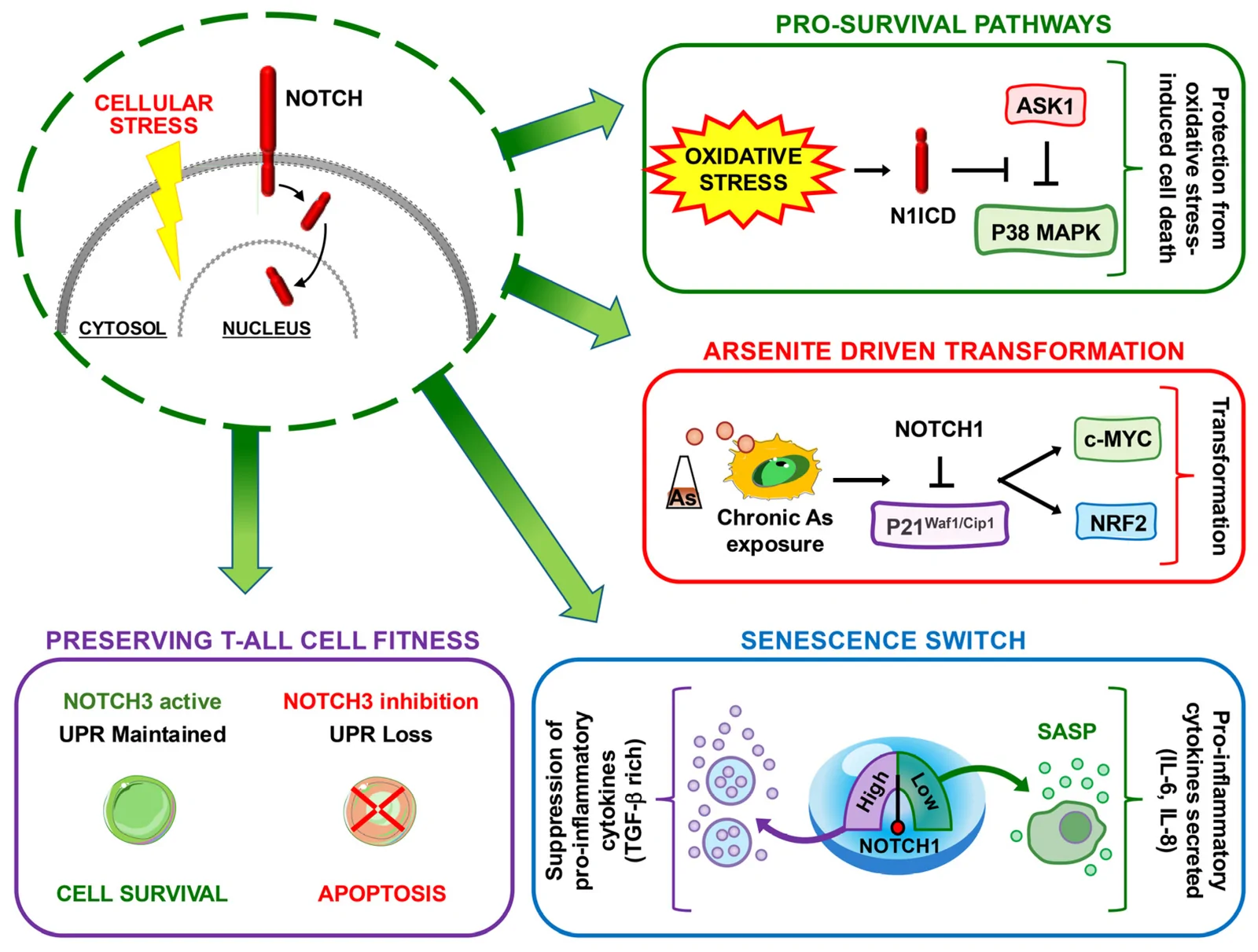
NOTCH1 orchestrates distinct stress-adaptive programs to determine cell fate. Upon cellular stress, N1ICD is engaged in context-specific adaptive circuits. Under oxidative stress, N1ICD inhibits the ASK1–p38 MAPK axis, protecting cells from oxidative stress-induced death. Chronic arsenite exposure restores NOTCH1 activity, which represses p21^Waf1/Cip1^ and cooperates with c-MYC and NRF2-dependent redox programs to promote malignant transformation. In T-ALL, active NOTCH3 sustains the unfolded protein response (UPR) and preserves leukemic cell fitness, whereas its inhibition disrupts UPR maintenance and promotes susceptibility to apoptosis. Independently, the nuclear NOTCH1 level governs a senescence switch. High levels of NOTCH1 promote a TGF-β-rich secretory phenotype with suppression of pro-inflammatory cytokines, whereas low levels of NOTCH1 favor the classical pro-inflammatory senescence-associated secretory phenotype (SASP; IL-6, IL-8).

If the DDR provides a paradigmatic example of how NOTCH1 activity can be differentially engaged according to cellular context, an equally important aspect concerns its role in shaping the biological states that emerge following stress exposure. Cellular stress does not inevitably lead to a single biological outcome. Depending on the nature, intensity and duration of the insult, cells may undergo apoptosis, differentiation, senescence, quiescence, regenerative activation, or long-term survival. Increasing evidence suggests that NOTCH1 is repeatedly engaged in these adaptive transitions ([29,30,31,32] and references therein). However, rather than functioning as a binary regulator that imposes a predefined response, NOTCH1 appears to operate as a highly context-dependent signaling node whose activity can be selectively activated, attenuated, or reconfigured according to the adaptive requirements imposed by each cellular state. One of the most compelling examples of this concept emerges from studies of cellular senescence. While senescence has traditionally been viewed as a stable endpoint characterized by irreversible proliferative arrest, recent findings indicate that senescent cells can adopt distinct functional states with markedly different biological properties. Hoare and colleagues demonstrated that oncogene-induced senescence is accompanied by dynamic fluctuations in NOTCH1 activity, which orchestrate a switch between two functionally distinct secretory programs [33]. Elevated NOTCH1 activity promotes a TGF-β-rich secretome while suppressing pro-inflammatory cytokine production, whereas subsequent reduction in NOTCH1 signaling permits the establishment of the classical inflammatory senescence-associated secretory phenotype (SASP) (Figure 2).

These findings indicate that NOTCH1 does not simply participate in the establishment of senescence but contributes to defining the functional identity of senescent cells by regulating how they interact with their surrounding microenvironment. The concept that NOTCH1 shapes distinct senescent states was further reinforced by the work of Teo and colleagues, who demonstrated that secondary senescence represents a molecularly distinct endpoint that requires Notch signaling rather than paracrine SASP signaling alone [34]. Importantly, secondary senescent cells displayed a transcriptional program characterized by a blunted secretory phenotype and the induction of fibrillar collagen genes, indicating that NOTCH1-dependent senescence is associated with a functional diversification of cellular responses rather than a uniform senescence program. These observations support the idea that NOTCH1 can contribute to the generation of alternative adaptive states following cellular stress, thereby increasing the spectrum of biological responses available to stressed tissues. A similarly dynamic regulation of NOTCH1 activity has been observed under conditions of cellular stress. Antila and colleagues demonstrated that multiple stress-associated stimuli induce SUMOylation of the NOTCH1 intracellular domain (N1ICD), resulting in attenuation of NOTCH1 transcriptional activity despite preserved nuclear localization [35]. Mechanistically, stress-induced SUMOylation promoted recruitment of HDAC4 to the NOTCH1 transcriptional complex, thereby repressing NOTCH1 target gene expression. These findings are particularly relevant because they demonstrate that stress does not necessarily activate or inhibit NOTCH1 signaling in a binary manner. Instead, cellular stress can selectively reprogram the transcriptional output of NOTCH1, allowing distinct biological responses to emerge from the same signaling pathway according to contextual demands. Evidence that NOTCH1 can actively support stress adaptation also derives from studies investigating oxidative stress responses. Mo and colleagues showed that N1ICD directly suppresses ASK1 signaling and inhibits activation of the downstream p38 MAPK stress-response cascade following oxidative injury [36] (Figure 2). As a consequence, NOTCH1 activation protects cells from oxidative stress-induced cell death, indicating that NOTCH1 can be utilized to preserve cellular viability under adverse conditions. A related stress-adaptive function of NOTCH1 was observed under chronic arsenite exposure, which restored NOTCH1 activity and promoted transformation through repression of p21^Waf1/Cip1^ and cooperation with c-MYC/NRF2-dependent redox programs [37] (Figure 2). Importantly, these findings extend the role of NOTCH1 beyond its classical functions in lineage specification and highlight its capacity to participate directly in stress-response networks that determine cellular survival. A comparable survival-oriented logic also extends to other Notch receptors. In T-ALL, for example, NOTCH3 sustains the unfolded protein response under proteotoxic stress, thereby preserving leukemic cell fitness; conversely, NOTCH3 inhibition disrupts this protective program and increases susceptibility to ER-stress-induced apoptosis [38] (Figure 2). Conversely, in other biological contexts NOTCH1 appears to facilitate tissue recovery and regenerative responses following injury. Studies of irradiation-induced bone marrow damage demonstrated that activation of canonical Notch signaling promotes hematopoietic recovery by enhancing proliferation and reducing apoptosis within hematopoietic progenitor populations [39]. In this context, Notch signaling contributes to restoration of tissue homeostasis following genotoxic injury rather than simply promoting survival of damaged cells. These findings further emphasize that the biological consequences of NOTCH1 activation are determined by the physiological context in which signaling occurs. Rather, NOTCH1 appears to function as an adaptive module that can be differentially used to stabilize or remodel distinct cellular states according to environmental demands. Depending on the biological context, Notch signaling may promote survival, facilitate regenerative responses, suppress inflammatory programs, reshape secretory phenotypes, or support the establishment of alternative senescent states. Such functional versatility suggests that the contribution of Notch signaling to tumor evolution may extend beyond its established roles in stemness and cell-fate determination. Instead, Notch signaling may represent one of the molecular systems through which cells dynamically adjust their phenotypic behavior in response to stress, thereby expanding the repertoire of adaptive strategies available during cancer progression.

## 4. Post-Translational and Epigenetic Regulation of Notch Signaling: Preserving Signaling Plasticity Through Dynamic Modulation

**Figure 3:**
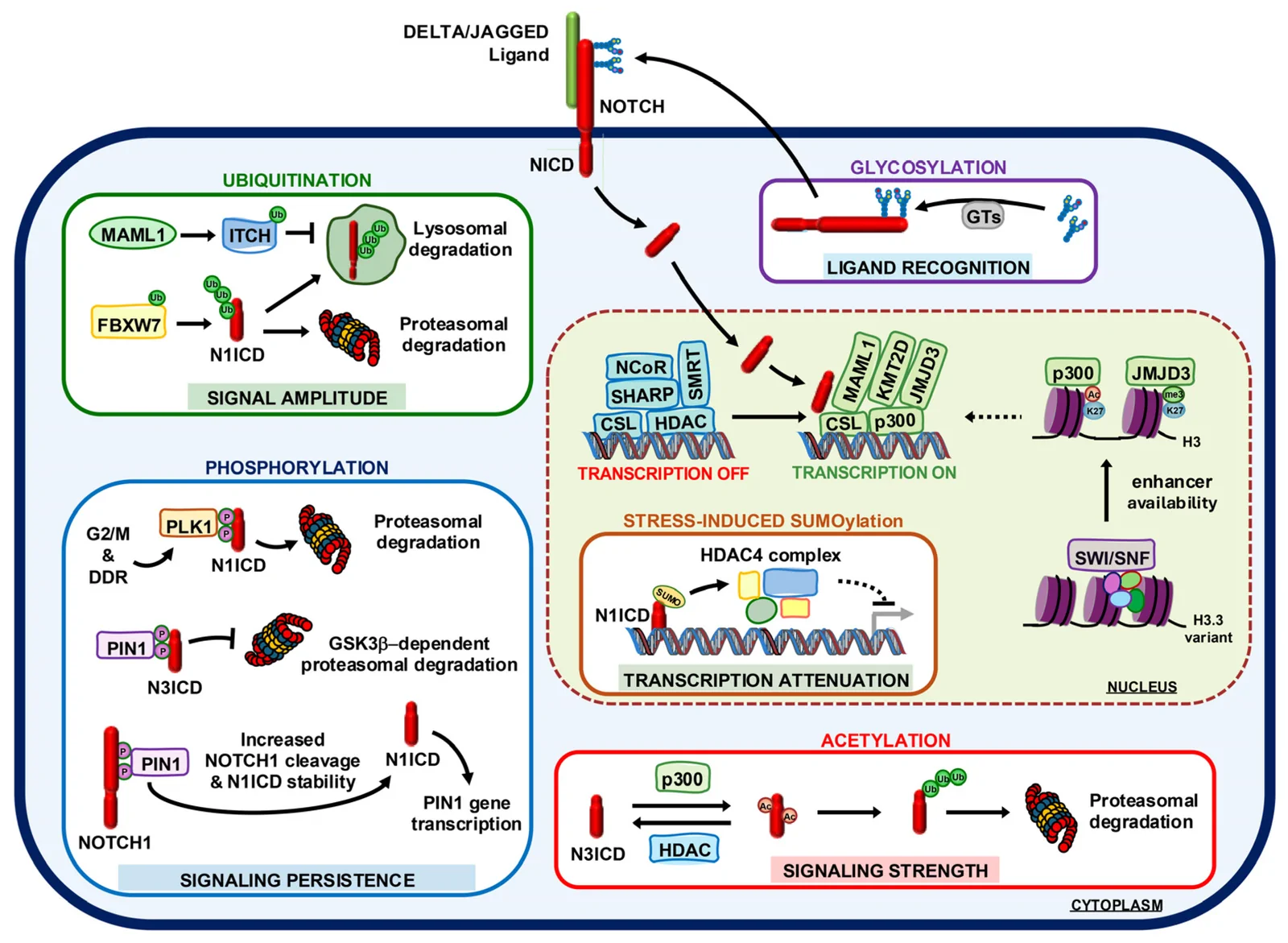
Mechanisms tuning Notch signaling plasticity. Schematic overview of the regulatory circuits that modulate NOTCH pathway activity. Glycosylation of the receptor, mediated by glycosyltransferases (GTs), along the endoplasmic reticulum/Golgi axis, contributes to ligand recognition and signaling strength. Downstream of receptor activation, cytosolic and nuclear modifications further shape pathway output. Ubiquitination regulates signal amplitude; MAML1 promotes ITCH self-ubiquitylation, thereby impairing ITCH-mediated lysosomal degradation of NOTCH1; and FBXW7-dependent ubiquitylation of N1ICD targets it for proteasomal degradation. Phosphorylation-dependent mechanisms tune signaling persistence. PLK1 integrates N1ICD regulation with G2/M progression and the DNA damage response via an FBXW7-dependent route, while PIN1 protects N3ICD from GSK3β-mediated proteasomal degradation. In addition, PIN1 binding promotes NOTCH1 cleavage and N1ICD stability, which in turn sustains PIN1 gene transcription, establishing a positive feedback loop. Acetylation of N3ICD by p300, counteracted by HDAC-mediated deacetylation, promotes its ubiquitination and proteasomal degradation, providing an additional layer of control over NOTCH3 signaling. In the nucleus, SWI/SNF-dependent chromatin remodeling and H3.3 incorporation promote enhancer availability favoring recruitment of the histone-modifying enzymes p300 and JMJD3, which respectively deposit H3K27ac and remove H3K27me3, thereby promoting assembly of the transcriptionally active RBPJ/CSL complex. Transcriptional output depends on the composition of the RBPJ/CSL complex. In the absence of NIICD, association with SHARP, NCoR/SMRT, and HDAC-containing complexes keeps target genes transcriptionally inactive (OFF), whereas the presence of N1ICD drives the recruitment of MAML1, p300, KMT2D and JMJD3 switching transcription ON. Under cellular stress, SUMOylation of nuclear N1ICD recruits an HDAC4-containing repressor complex that attenuates transcription of NOTCH target genes.

The remarkable diversity of biological outputs associated with Notch signaling raises an important mechanistic question: how can a pathway built upon a relatively simple signaling architecture (Figure 1 and Figure 2) generate such distinct and often opposing cellular responses? Increasing evidence suggests that a substantial part of this versatility resides in the extensive network of post-translational modifications and epigenetic mechanisms that continuously regulate Notch signaling activity at multiple levels. Importantly, these regulatory processes do not simply control receptor abundance. Rather, they influence receptor trafficking, protein stability, transcriptional activity, subcellular localization, ligand responsiveness, and the composition of the nuclear transcriptional complex, thereby shaping the qualitative output of the pathway according to cellular context [40,41,42,43]. Among the most extensively studied mechanisms are ubiquitin-dependent regulatory circuits. Ubiquitination of NOTCH receptors and ligands controls multiple steps of pathway activation, including receptor trafficking, endosomal sorting, ligand internalization, transcriptional competence, and proteasomal degradation [44,45]. Importantly, ubiquitination does not invariably result in pathway silencing. Depending on the specific ubiquitin ligase involved and the cellular compartment in which modification occurs, ubiquitination may either attenuate signaling or facilitate productive receptor activation by controlling intracellular trafficking and receptor turnover. These observations indicate that ubiquitin-dependent mechanisms function primarily as modulators of signaling amplitude and duration rather than as simple on–off switches. Recent evidence further highlights the complexity of ubiquitin-mediated regulation of Notch signaling. It has been shown that the transcriptional co-activator MAML1 can regulate NOTCH1 stability through a non-canonical mechanism involving the E3 ubiquitin ligase ITCH. Mechanistically, MAML1 interacts with ITCH and promotes its K63-linked self-ubiquitylation, thereby impairing ITCH-dependent ubiquitylation and degradation of NOTCH1. As a consequence, increased MAML1 expression stabilizes NOTCH1 and enhances pathway activity [13] (Figure 3).

Together with previous evidence placing MAML1 within broader oncogenic and signaling regulatory networks [46], these findings illustrate how components traditionally viewed as transcriptional regulators may also influence Notch signaling at the post-translational level, further extending the repertoire of mechanisms capable of modulating pathway availability without directly altering receptor expression. A similar principle emerges from studies investigating phosphorylation-dependent regulation of NOTCH1. Phosphorylation influences receptor activity through multiple mechanisms, including regulation of protein–protein interactions, nuclear localization, transcriptional competence, and receptor degradation. Phosphorylation-dependent recruitment of the E3 ubiquitin ligase FBXW7 contributes to N1ICD turnover and limits excessive pathway activation (Figure 3) [47]. However, rather than abolishing signaling, these mechanisms continuously adjust the intensity and persistence of NOTCH1 activity, thereby preserving signaling responsiveness while preventing uncontrolled activation. An additional level of complexity is provided by the interaction between NOTCH1 and PLK1. NOTCH1 has been identified as a direct target of PLK1 and, interestingly, the functional relationship between these proteins changes dynamically during cell-cycle progression and genotoxic stress. PLK1 contributes to NOTCH1 regulation during G2/M progression, whereas DNA damage-induced inhibition of PLK1 preserves NOTCH1 expression while simultaneously restraining mitotic entry (Figure 3). Furthermore, arsenite-induced genotoxic stress triggers a PLK1-dependent signaling response capable of antagonizing NOTCH1 participation in DNA damage checkpoint regulation [9]. These observations suggest that phosphorylation-dependent mechanisms do not simply regulate NOTCH1 abundance but can actively reshape the biological function of the pathway according to cellular requirements, allowing the same signaling module to support distinct and sometimes opposing adaptive responses. Additional evidence supporting this concept derives from PIN1, which emerged as an important regulator of NOTCH1 signaling through its ability to recognize phosphorylated Ser/Thr-Pro motifs and modify the conformational state of its substrates. Rustighi and colleagues demonstrated that PIN1 directly binds the Ser/Thr-Pro-rich region of NOTCH1 in a phosphorylation-dependent manner, enhancing γ-secretase-dependent NOTCH1 cleavage and increasing the levels of activated N1ICD in breast cancer cells [48]. Importantly, this interaction was shown to establish a positive regulatory circuit, because activated NOTCH1 promotes PIN1 transcription, thereby reinforcing NOTCH1 pathway activation and supporting oncogenic growth (Figure 3). Subsequent work from the same group extended this concept to normal and cancer stem cells of the breast, showing that PIN1 sustains Notch signaling by protecting NOTCH1 and NOTCH4 from FBXW7α-dependent proteasomal degradation [49]. Functionally, this PIN1-dependent stabilization of Notch activity promoted stem-cell self-renewal, tumor growth and metastatic potential, indicating that PIN1 can preserve NOTCH1-dependent adaptive states by modulating receptor stability rather than by permanently fixing pathway output. These findings place the PIN1–NOTCH1 axis as a central example of how phosphorylation-dependent post-translational regulation can amplify, stabilize and functionally redirect NOTCH1 signaling in cancer. Studies on NOTCH3 provide complementary evidence that the same regulatory logic can extend to other NOTCH receptors in specific cellular contexts. In T-ALL, PIN1 was shown to bind and stabilize NOTCH3, increasing N3ICD levels and promoting NOTCH3-dependent invasive properties, thereby identifying a NOTCH3–PIN1 regulatory axis relevant to leukemic progression [50]. Consistently, in high-grade serous ovarian cancer, PIN1 binding protects NOTCH3 from GSK3β-dependent degradation, resulting in enhanced receptor stability and sustained signaling activity under platinum-induced selective pressure [10] (Figure 3). Additional NOTCH3-focused studies also indicate that post-translational mechanisms control receptor availability and signaling output. Acetylation of NOTCH3 by p300 and deacetylation by HDAC1 regulate receptor stability and promote ubiquitin/proteasome-dependent degradation [51], whereas c-CBL-dependent ubiquitination of NOTCH3 is influenced by subcellular localization and contributes to the control of NOTCH3 signaling in leukemic cells [52] (Figure 3). Together, these observations reinforce the notion that post-translational regulation represents a general mechanism through which Notch signaling is dynamically tuned across receptor paralogues and cellular contexts and illustrates how PTMs can regulate the duration and availability of Notch signaling without requiring permanent pathway activation. Perhaps the most illustrative example of adaptive modulation derives from stress-induced SUMOylation of N1ICD. Antila and colleagues demonstrated that multiple forms of cellular stress promote SUMO conjugation of N1ICD within the nucleus, resulting in reduced transcriptional activation of canonical Notch target genes [35]. Notably, SUMOylation does not prevent N1ICD nuclear accumulation, nor does it disrupt assembly of the NOTCH transcriptional complex. Instead, SUMOylated N1ICD remains associated with the transcriptional machinery while recruiting HDAC4-containing repressive complexes that dampen transcriptional output (Figure 3). Consequently, stress-induced SUMOylation acts to buffer NOTCH activity rather than eliminate it. This distinction may be biologically significant, as complete pathway silencing would remove the possibility of rapidly re-engaging NOTCH-dependent adaptive programs should environmental conditions change. Importantly, the adaptive regulation of Notch signaling is not restricted to modifications directly targeting the receptor itself. Increasing evidence indicates that chromatin remodeling and epigenetic regulatory mechanisms provide an additional layer of control capable of modulating pathway output without necessarily extinguishing signaling competence. Although these mechanisms do not constitute post-translational modifications in the strict sense, they profoundly influence the accessibility of NOTCH-responsive loci and the ability of cells to engage Notch-dependent transcriptional programs. In this context, regulation of chromatin architecture may serve as a mechanism to constrain or facilitate Notch signaling while preserving the possibility of rapid pathway reactivation in response to changing environmental conditions. Consistent with this view, Pillidge and Bray demonstrated that SWI/SNF-dependent chromatin remodeling is required to maintain accessibility of Notch-responsive enhancers and support productive transcriptional responses downstream of pathway activation [53]. Their work revealed that Notch activation is associated with increased nucleosome turnover and enhanced incorporation of the histone variant H3.3 at responsive loci, whereas disruption of the Brahma SWI/SNF complex markedly impaired enhancer accessibility and recruitment of the Notch transcriptional machinery. These findings indicate that chromatin remodeling could function as an active determinant of Notch signaling competence itself. A complementary level of regulation operates through the RBPJ/CSL transcriptional complex. Giaimo and colleagues proposed that RBPJ functions as a dynamic molecular switch capable of recruiting either activating or repressive chromatin-modifying complexes depending on signaling status [54] (Figure 3). In the absence of NICD, RBPJ associates with SHARP, NCoR/SMRT, and HDAC-containing complexes that promote transcriptional repression. Upon pathway activation, these complexes are displaced by NICD-dependent co-activator assemblies containing p300 and KMT2D-associated chromatin modifiers. Importantly, this transition does not necessarily represent a binary shift between active and inactive chromatin states. Rather, transcriptionally responsive chromatin configurations appear capable of preserving target-gene competence while limiting transcriptional output, thereby allowing rapid transitions between repression and activation according to cellular requirements. Further support for this model derives from studies linking Notch signaling to histone-modifying enzymes. Tottone and colleagues demonstrated that both NOTCH1 and NOTCH3 signaling contribute to the recruitment of JMJD3 and p300 at the NOTCH3 locus, thereby maintaining permissive H3K27 chromatin states and sustaining its transcription in T-ALL [55]. Pharmacological inhibition of either JMJD3 or p300 reduced expression of NOTCH receptors themselves together with canonical downstream targets, indicating that chromatin modifications contribute not only to pathway activation but also to the maintenance of signaling competence. These findings suggest that epigenetic regulation participates in preserving a transcriptional landscape permissive for future Notch-dependent responses rather than simply controlling pathway activation at a given moment. Additional layers of regulation further reinforce this concept. Glycosylation of NOTCH receptors profoundly influences ligand recognition, receptor folding, trafficking, and signaling strength without necessarily altering receptor abundance [56,57]. Likewise, regulation of CSL/RBPJ availability by stress-responsive pathways provides an additional mechanism through which NOTCH transcriptional output can be selectively attenuated under conditions of cellular stress [58]. Collectively, these observations indicate that cells possess multiple regulatory nodes capable of fine-tuning Notch signaling at different levels without completely abolishing pathway activity. Taken together, these findings suggest that regulation of Notch signaling serves a broader function than the simple control of receptor stability or pathway activation. Rather, post-translational and epigenetic mechanisms cooperate to maintain Notch signaling in a dynamically adjustable state, preventing both irreversible activation and complete suppression. From this perspective, attenuation of NOTCH activity may represent a strategy to temporarily constrain specific signaling outputs while preserving the capacity to rapidly reactivate alternative adaptive programs when environmental conditions demand it. Such a regulatory architecture is particularly important in the context of tumor plasticity, where maintaining signaling flexibility might be more advantageous than enforcing a fixed cellular state. Consequently, these regulatory mechanisms may constitute a central component of the molecular framework through which cancer cells preserve the adaptive potential associated with Notch signaling while continuously reshaping its biological output according to changing selective pressures.

## 5. Notch Signaling as a Driver of Adaptive Cell-State Transitions

The ability of tumor cells to continuously adapt to changing environmental conditions relies on their capacity to preserve the ability to adopt distinct cellular states. While cancer evolution was historically interpreted through hierarchical models in which specific cellular populations occupied fixed functional positions, increasing evidence suggests that many tumor-associated phenotypes are dynamically acquired and reversible [59]. Among these, the acquisition of stem-like properties represents one of the best-characterized manifestations of cellular plasticity. Recent conceptual frameworks have challenged the traditional view of cancer stem cells (CSCs) as a rare and static population, proposing instead that stemness represents a dynamic cellular state that can emerge, disappear, and re-emerge according to environmental and therapeutic pressures [2]. Within this model, transitions between stem-like and non-stem-like phenotypes constitute a fundamental component of tumor adaptation and contribute to intratumoral heterogeneity, therapeutic resistance, and long-term disease progression. This evolving view of cancer stemness closely parallels recent advances in normal stem cell biology. Studies of physiological tissues have demonstrated that stemness cannot be reduced to a rigid cellular identity but rather reflects a collection of functional properties, including self-renewal, plasticity, dependence on niche-derived signals, and the ability to preserve long-term regenerative potential [60]. Importantly, these properties are not immutable and can be dynamically regulated by environmental cues. Similar principles appear to operate in cancer, where stem-like phenotypes are increasingly recognized as adaptive cellular states rather than permanently defined populations. Within this context, Notch signaling could occupy a particularly prominent role. Canonically, NOTCH regulates stem cell maintenance, lineage commitment, and tissue regeneration across multiple developmental systems. However, the association between NOTCH activity and cancer stemness suggests a broader role extending beyond the preservation of developmental hierarchies. Rather than simply maintaining stem-cell identity, Notch signaling appears to participate in the molecular circuitry that enables tumor cells to acquire and stabilize stem-like phenotypes when such states become advantageous. Consistent with this view, aberrant NOTCH activation has been implicated in the maintenance of cancer stem-like populations across multiple solid and hematological malignancies, where it contributes to self-renewal, tumor initiation, cellular heterogeneity, and resistance to therapy [61]. Importantly, recent studies suggest that cancer stemness itself may represent an adaptive response to selective pressures rather than a constitutive property of a predefined cellular subset. Hypoxia, metabolic stress, inflammation, therapeutic exposure, and microenvironmental remodeling have all been shown to promote the emergence of stem-like phenotypes through extensive transcriptional, epigenetic, and metabolic reprogramming [62,63]. Under these conditions, acquisition of stem-like properties provides a selective advantage by increasing cellular fitness, promoting survival under adverse conditions, and expanding the repertoire of potential biological responses available to the tumor population. Consistent with this adaptive role, NOTCH3 signaling has been implicated in the maintenance of cancer stem-like properties and chemoresistance in ovarian cancer [64]. NOTCH-dependent therapeutic resistance has also been associated with CSC- and epithelial–mesenchymal transition (EMT)-related phenotypes, drug-efflux mechanisms, and tumor–microenvironment interactions [65]. Collectively, these observations support the view that stemness might constitute one of the adaptive states through which cancer cells preserve evolutionary flexibility during tumor progression. Several observations further support this perspective. Cancer stem-like cells exhibit enhanced resistance to genotoxic stress, increased capacity for metabolic adaptation, and improved tolerance to microenvironment constraints. These characteristics closely overlap with the broader adaptive programs discussed above, including DNA damage responses, stress adaptation, and cellular reprogramming. Notably, many of the molecular pathways that sustain stem-like states, including NOTCH, WNT, Hedgehog, inflammatory signaling, and epigenetic remodeling networks, are similarly engaged during cellular responses to environmental stress, suggesting that stemness may emerge as part of a larger adaptive strategy rather than as an isolated biological phenomenon [2]. Further evidence supporting the dynamic nature of stem-like states derives from studies demonstrating bidirectional interconversion between stem-like and non-stem-like populations. Rather than representing a terminal differentiation hierarchy, many tumors exhibit continuous phenotypic flux in which differentiated cells can reacquire stem-cell properties following environmental perturbation or therapeutic challenge. This plasticity is increasingly recognized as a major determinant of tumor progression and treatment failure [66]. Within this framework, Notch signaling may not function as a dedicated regulator of stemness *per se*. Instead, Notch signaling appears to be repeatedly engaged during processes of cellular reprogramming, where it contributes to the stabilization of adaptive phenotypic states according to contextual demands.

A particularly illustrative example of this concept emerges from studies of hematopoietic stem-cell biology, where distinct configurations of Notch signaling have been shown to regulate stem-cell state transitions. In embryonic hematopoiesis, NOTCH1–JAG1 cis-interactions preserve immature HSC phenotypes by restraining differentiation programs, whereas in adult hematopoiesis age-associated alterations in JAG2-dependent Notch signaling promote expansion of myeloid-biased stem-cell populations and impair regenerative potential [67,68]. These findings indicate that subtle modulation of Notch signaling can influence the balance between alternative stem-cell states without necessarily abolishing stem-cell identity itself. Such observations reinforce the concept that Notch signaling functions less as a binary determinant of cellular fate and more as a regulator of transitions between functionally distinct cellular states. Collectively, these findings suggest that the relationship between Notch signaling and cancer stemness should not be interpreted exclusively through the classical paradigm of stem-cell maintenance. Rather, stem-like phenotypes may represent one manifestation of a broader adaptive capacity that allows tumor cells to dynamically explore alternative cellular states. In this context, Notch signaling emerges not as the origin of cellular plasticity itself, but as one of the molecular systems repeatedly exploited by tumor cells to facilitate state transitions, preserve phenotypic flexibility, and maintain the adaptive potential required for long-term tumor evolution.

## 6. Notch Signaling as an Adaptive Platform in Cancer Evolution

**Figure 4:**
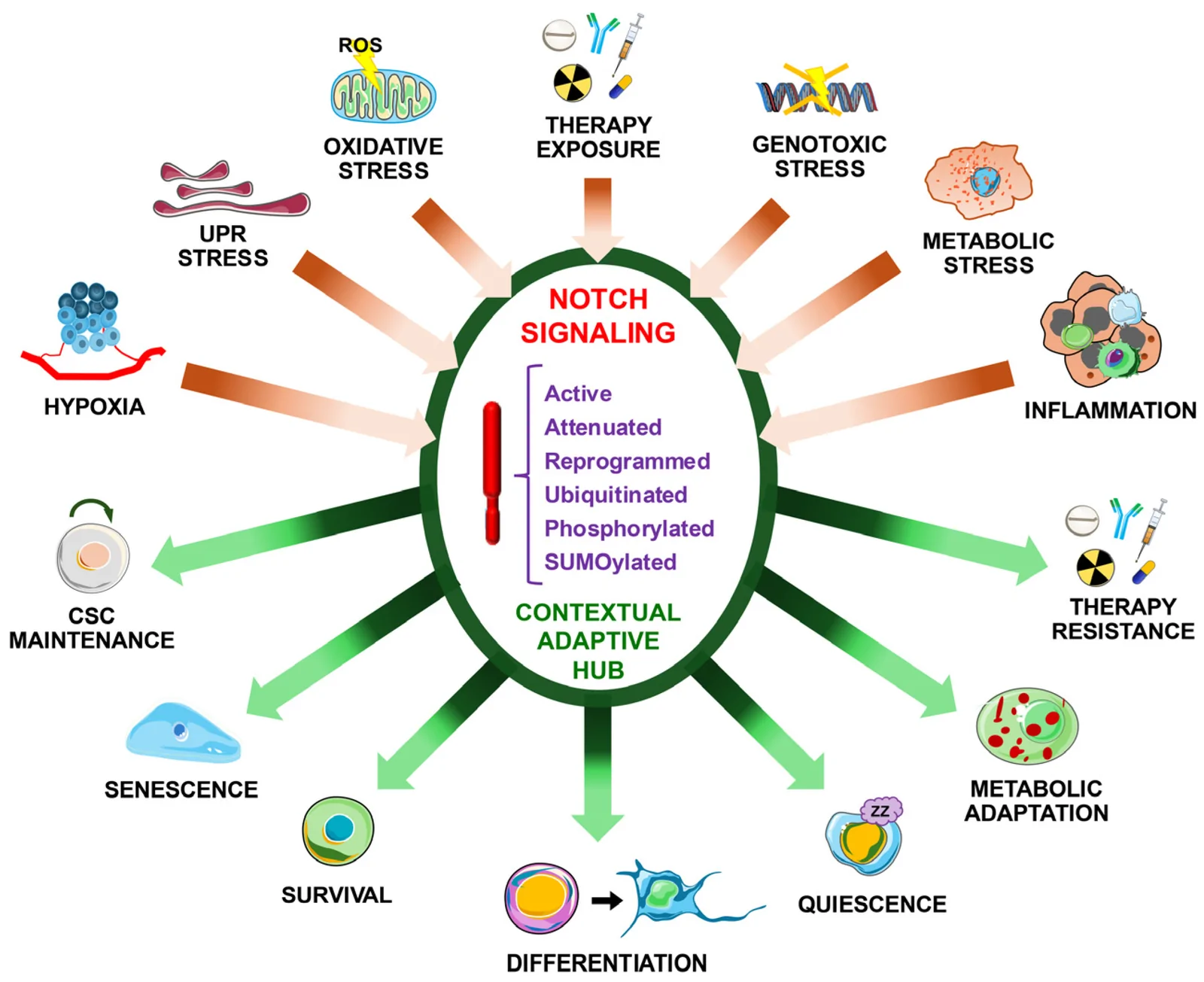
Notch signaling as a versatile regulatory platform for cellular adaptation in cancer. The proposed model summarizes evidence linking Notch signaling to cellular response under different environmental and therapeutic pressures. Thus, environmental pressures, including hypoxia, proteotoxic/UPR-related stress, oxidative stress, therapy exposure, genotoxic stress, metabolic stress, and inflammation, have been associated with changes in Notch signaling, which integrates these cues by adopting distinct signaling states (active, attenuated or reprogrammed), shaped by post-translational modifications, including ubiquitination, phosphorylation and SUMOylation. Depending on the input received and the cellular context, Notch signaling might contribute to directing tumor cells toward several non-exclusive phenotypic outcomes, including CSC maintenance, senescence, survival, differentiation, quiescence, metabolic adaptation, and therapy resistance. These outcomes could represent reversible states that Notch signaling might engage according to the distinct pressures acting on cancer cells rather than fixed endpoints.

Tumor plasticity could be defined as the capacity of cancer cells to continuously reconfigure their phenotypic state in response to changing environmental conditions. In this context, stemness represents one possible adaptive configuration among several others, including differentiation, metabolic adaptation, quiescence, and stress-response programs. The adoption of these phenotypes is governed by regulatory mechanisms that allow tumor cells to selectively engage the biological state most compatible with survival and fitness under a given set of conditions. Similarly, the biological significance of Notch signaling in cancer extends beyond its established roles in cell-fate determination, stem-cell maintenance and lineage specification. Although these developmental functions undoubtedly contribute to tumor biology, accumulating evidence indicates that they do not fully explain the remarkably broad range of biological contexts in which Notch signaling becomes engaged. Rather, a recurring theme emerging from diverse experimental systems is the association of Notch activity with cellular states characterized by environmental perturbation, cellular stress, and adaptive responses. This principle is particularly evident in the context of DNA damage responses. Studies on *D. melanogaster* first identified components of the Notch pathway among factors required for survival following genotoxic stress [20], while investigations in *C. elegans* demonstrated that Notch-dependent cellular states display distinct competencies to activate ATM-mediated responses following irradiation [21,22]. In mammalian systems, NOTCH1 has been shown to exert either positive or negative effects on DNA damage signaling depending on cellular context, influencing ATM activity, checkpoint responses, proliferative recovery, and survival under genotoxic stress [9,21]. Collectively, these observations argue against a fixed role of Notch in DNA damage responses and instead support a model in which Notch signaling is differentially utilized according to the adaptive requirements imposed by cellular stress. A similar principle emerges from studies investigating senescence and stress-induced cellular reprogramming. Dynamic fluctuations in Notch activity have been shown to regulate distinct senescent states and secretory phenotypes, influencing how stressed cells communicate with and remodel their surrounding microenvironment [33,34]. Likewise, stress-responsive mechanisms such as SUMOylation, oxidative stress signaling, and inflammatory pathways can selectively reshape Notch transcriptional output without completely abolishing pathway activity [35,36]. These findings suggest that Notch signaling remains functionally available even under conditions that attenuate its activity, thereby preserving the possibility of engaging alternative adaptive programs when environmental conditions change. Importantly, the molecular mechanisms regulating Notch signaling appear particularly compatible with such a model. Ubiquitination, phosphorylation, SUMOylation, chromatin remodeling, and transcriptional regulation through RBPJ/CSL complexes do not simply switch the pathway on or off. Rather, they might also continuously modulate signaling intensity, transcriptional competence, receptor stability, and target-gene accessibility [35,45,53,54,55]. Such regulatory architectures suggest that preserving signaling flexibility may be more advantageous than enforcing complete pathway activation or irreversible silencing. From an evolutionary perspective, maintaining Notch signaling in a dynamically adjustable state may provide tumor cells with access to a broader repertoire of adaptive responses. Within this framework, the relationship between Notch signaling and cancer stemness can also be reinterpreted. Traditionally, Notch has been viewed as a central regulator of stem-cell maintenance, and numerous studies have demonstrated its contribution to the establishment and persistence of cancer stem-like populations across multiple malignancies [61]. However, stemness might be described as a dynamic and reversible cellular state rather than a fixed cellular identity [2,66]. From this perspective, the recurrent involvement of Notch signaling in stem-like phenotypes may not necessarily indicate that stemness constitutes its primary biological function. Instead, acquisition of stem-like properties may represent one of several adaptive states that tumor cells can access through context-dependent engagement of Notch signaling. Indeed, many of the biological properties commonly attributed to cancer stemness including resistance to DNA damage, tolerance to oxidative stress, metabolic flexibility, long-term survival, and enhanced responsiveness to environmental cues overlap substantially with the adaptive programs discussed above. The selective emergence of stem-like phenotypes following hypoxia, therapeutic exposure, inflammatory signaling, or metabolic stress suggests that stemness itself may represent a context-dependent adaptive solution rather than a constitutive cellular trait [62,63,69]. In this scenario, Notch signaling would not function primarily to generate stemness, but rather to facilitate transitions toward cellular states that maximize fitness under specific environmental constraints. Such an interpretation may also help reconcile the apparent functional contradictions that have historically characterized the Notch field. The ability of Notch signaling to promote proliferation in some contexts, induce differentiation in others, support senescence-associated programs, regulate DNA damage responses, sustain stem-like states, or contribute to therapeutic resistance may not reflect unrelated biological functions. Rather, these diverse outcomes may represent different manifestations of a common adaptive principle. Depending on the selective pressures operating within a given tumor ecosystem, Notch signaling can be differentially engaged, attenuated, or rewired to support the cellular state most compatible with survival and evolutionary success (Figure 4).

## 7. Conclusions

Tumor evolution arises from stochastic cellular variation, yet the trajectory a tumor follows is shaped by the selective pressures imposed by its ecosystem. Within this context, the evidence reviewed here supports a conceptual shift in the interpretation of NOTCH biology in cancer. Notch signaling does not function as a fixed oncogene or tumor suppressor, but rather as a dynamic platform whose output is determined by the ecosystem in which the tumor resides. Even in contexts in which Notch signaling appears to exert tumor-suppressive functions, maintaining its tunable plasticity may be more advantageous to the tumor than its irreversible loss, preserving the capacity to re-engage the pathway should selective conditions change. We therefore propose that, rather than being defined by a fixed oncogenic or tumor-suppressive identity, Notch signaling may constitute a paradigmatic adaptive platform whose principal contribution to cancer progression lies not in controlling a single phenotypic output such as stemness, therapy resistance, or senescence but in preserving the phenotypic flexibility that enables tumor cells to respond to changing selective conditions. Within this framework, these phenotypic states should not be viewed as independent outputs of Notch signaling, but as context-dependent adaptive manifestations of the capacity of tumor cells to exploit Notch-dependent regulatory networks according to environmental, metabolic, genotoxic, inflammatory, and therapeutic demands. Thus, the principal contribution of Notch signaling to cancer progression may lie not in the control of any specific biological program, but in its capacity to preserve the phenotypic flexibility that underlies tumor adaptation and evolution (Figure 4). This perspective raises the possibility that defining the extent to which context-dependent Notch signaling contributes to the maintenance of tumor cell phenotypic flexibility may have important therapeutic implications. In particular, establishing whether Notch-dependent regulatory mechanisms enable tumor cells to access alternative adaptive states under therapeutic pressure could provide a rationale for strategies aimed at constraining this adaptive capacity. In molecularly defined tumor contexts, such strategies might complement the targeting of oncogenic dependencies that sustain tumor fitness, potentially limiting the emergence of adaptive responses that allow tumor cells to escape therapeutic pressure.

## Data Availability

No new data were created or analyzed in this study. Data sharing is not applicable to this article.

## References

[1] Lourenço F.C., Sadien I.D., Wong K., Adler S., Sawle A., Santana L.S., Hazelwood L., Giavara G., Nicholson A.M., Eldridge M.D. (2026). Decay of driver mutations shapes the landscape of intestinal transformation. Nature.

[2] Loh J.J., Ma S. (2024). Hallmarks of cancer stemness. Cell Stem Cell.

[3] Artavanis-Tsakonas S., Matsuno K., Fortini M.E. (1995). Notch signaling. Science.

[4] Kopan R., Ilagan M.X. (2009). The canonical notch signaling pathway: Unfolding the activation mechanism. Cell.

[5] Giuli M.V., Giuliani E., Screpanti I., Bellavia D., Checquolo S. (2019). Notch signaling activation as a hallmark for triple-negative breast cancer subtype. J. Oncol..

[6] Zhdanovskaya N., Firrincieli M., Lazzari S., Pace E., Scribani Rossi P., Felli M.P., Talora C., Screpanti I., Palermo R. (2021). Targeting notch to maximize chemotherapeutic benefits: Rationale, advanced strategies, and future perspectives. Cancers.

[7] Koch U., Radtke F. (2007). Notch and cancer: A double-edged sword. Cell. Mol. Life Sci. CMLS.

[8] Sun W., Gaykalova D.A., Ochs M.F., Mambo E., Arnaoutakis D., Liu Y., Loyo M., Agrawal N., Howard J., Li R. (2014). Activation of the notch pathway in head and neck cancer. Cancer Res..

[9] De Blasio C., Zonfrilli A., Franchitto M., Mariano G., Cialfi S., Verma N., Checquolo S., Bellavia D., Palermo R., Benelli D. (2019). Plk1 targets notch1 during DNA damage and mitotic progression. J. Biol. Chem..

[10] Giuli M.V., Mancusi A., Natiello B., Di Cristofano S., Reali R., Pignataro M.G., D’Andrea D., Di Magno L., Nicoletti C., Giorgi A. (2026). Prolyl-isomerase pin1 drives platinum resistance by regulating notch3 stability and function in ovarian cancer. J. Exp. Clin. Cancer Res..

[11] Cialfi S., Palermo R., Manca S., Checquolo S., Bellavia D., Pelullo M., Quaranta R., Dominici C., Gulino A., Screpanti I. (2013). Glucocorticoid sensitivity of t-cell lymphoblastic leukemia/lymphoma is associated with glucocorticoid receptor-mediated inhibition of notch1 expression. Leukemia.

[12] Real P.J., Tosello V., Palomero T., Castillo M., Hernando E., de Stanchina E., Sulis M.L., Barnes K., Sawai C., Homminga I. (2009). Gamma-secretase inhibitors reverse glucocorticoid resistance in t cell acute lymphoblastic leukemia. Nat. Med..

[13] Zema S., Di Fazio F., Pelullo M., Di Savino S., Cerbelli B., Leopizzi M., Di Magno L., Nicoletti C., Peruzzi G., D’Andrea D. (2026). Maml1 drives notch and hedgehog oncogenic pathways by inhibiting itch activity in triple-negative breast cancer. Cell Death Differ..

[14] Al-Khreisat M.J., Abdulsahib W.K., Jasim I.K., Malathi H., Nayak P.P., Anand D.A., Mukherjee G., Sinha A., Ruziyev O. (2025). Targeting cancer stem cell plasticity and tumor microenvironment crosstalk: A comprehensive review. Discov. Oncol..

[15] Kulus M., Marchel M., Knopik-Skrocka A., Data K., Domagała D., Bednarska W., Mozdziak P., Kempisty B. (2026). Metabolic reprogramming and plasticity of cancer stem cells. Front. Cell Dev. Biol..

[16] Nagai H., Nakajima Y.I. (2026). Epithelial cell plasticity in metazoans: Evolutionary insights into roles and mechanisms. Semin. Cell Dev. Biol..

[17] Shepley B.R., Bain A.R. (2024). Is notch1 a neglected vascular mechanosensor?. Physiol. Rev..

[18] Singh T., Jacobs K.A., Polacheck W.J., Kutys M.L. (2026). The notch1 intracellular domain orchestrates mechanotransduction of fluid shear stress. Life Sci. Alliance.

[19] Yang T., Jiang Q., Tan D., Yang H., Shi Y., Zhou J., Li Y., Liu C., Li L., Yao X. (2026). Jagged1-notch1/foxo1 signaling crosstalk regulates txnip/nlrp3 inflammasome to alleviate lung inflammation. Cell. Signal..

[20] Ravi D., Wiles A.M., Bhavani S., Ruan J., Leder P., Bishop A.J. (2009). A network of conserved damage survival pathways revealed by a genomic rnai screen. PLoS Genet..

[21] Vermezovic J., Adamowicz M., Santarpia L., Rustighi A., Forcato M., Lucano C., Massimiliano L., Costanzo V., Bicciato S., Del Sal G. (2015). Notch is a direct negative regulator of the DNA-damage response. Nat. Struct. Mol. Biol..

[22] Hoffman S., Martin D., Meléndez A., Bargonetti J. (2014). *C. elegans* cep-1/p53 and bec-1 are involved in DNA repair. PLoS ONE.

[23] Katarkar A., Bottoni G., Clocchiatti A., Goruppi S., Bordignon P., Lazzaroni F., Gregnanin I., Ostano P., Neel V., Dotto G.P. (2020). Notch1 gene amplification promotes expansion of cancer associated fibroblast populations in human skin. Nat. Commun..

[24] Bernardo S., Brunet L., Thomas Q.D., Bracquemond D., Bouclier C., Colomb M., Mancini M., Fabbrizio E., Santos A., Rasamizafy S.F. (2026). Notch1 intracellular domain stabilization by mdm2 plays a major role in nsclc response to platinum. EMBO Mol. Med..

[25] Wang J., Wakeman T.P., Lathia J.D., Hjelmeland A.B., Wang X.F., White R.R., Rich J.N., Sullenger B.A. (2010). Notch promotes radioresistance of glioma stem cells. Stem Cells.

[26] Pan L., Lemieux M.E., Thomas T., Rogers J.M., Lipper C.H., Lee W., Johnson C., Sholl L.M., South A.P., Marto J.A. (2020). Ier5, a DNA damage response gene, is required for notch-mediated induction of squamous cell differentiation. eLife.

[27] Thakur V., Thakur V.S., Wang D., de Freitas J.T., Bianchi A., Nivelo L.A., Umland O., Welford S.M., Bedogni B. (2025). Co-inhibition of notch1 and chk1 triggers genomic instability and melanoma cell death increasing the lifespan of mice bearing melanoma brain metastasis. J. Exp. Clin. Cancer Res. CR.

[28] Miao K., Lei J.H., Valecha M.V., Zhang A., Xu J., Wang L., Lyu X., Chen S., Miao Z., Zhang X. (2020). Notch1 activation compensates brca1 deficiency and promotes triple-negative breast cancer formation. Nat. Commun..

[29] Bigas A., Espinosa L. (2018). The multiple usages of notch signaling in development, cell differentiation and cancer. Curr. Opin. Cell Biol..

[30] Bray S.J., Bigas A. (2025). Modes of notch signalling in development and disease. Nat. Rev. Mol. Cell Biol..

[31] Roy M., Pear W.S., Aster J.C. (2007). The multifaceted role of notch in cancer. Curr. Opin. Genet. Dev..

[32] Schwanbeck R., Martini S., Bernoth K., Just U. (2011). The notch signaling pathway: Molecular basis of cell context dependency. Eur. J. Cell Biol..

[33] Hoare M., Ito Y., Kang T.W., Weekes M.P., Matheson N.J., Patten D.A., Shetty S., Parry A.J., Menon S., Salama R. (2016). Notch1 mediates a switch between two distinct secretomes during senescence. Nat. Cell Biol..

[34] Teo Y.V., Rattanavirotkul N., Olova N., Salzano A., Quintanilla A., Tarrats N., Kiourtis C., Müller M., Green A.R., Adams P.D. (2019). Notch signaling mediates secondary senescence. Cell Rep..

[35] Antila C.J.M., Rraklli V., Blomster H.A., Dahlström K.M., Salminen T.A., Holmberg J., Sistonen L., Sahlgren C. (2018). Sumoylation of notch1 represses its target gene expression during cell stress. Cell Death Differ..

[36] Mo J.S., Yoon J.H., Ann E.J., Ahn J.S., Baek H.J., Lee H.J., Kim S.H., Kim Y.D., Kim M.Y., Park H.S. (2013). Notch1 modulates oxidative stress induced cell death through suppression of apoptosis signal-regulating kinase 1. Proc. Natl. Acad. Sci. USA.

[37] Cialfi S., Palermo R., Manca S., De Blasio C., Vargas Romero P., Checquolo S., Bellavia D., Uccelletti D., Saliola M., D’Alessandro A. (2014). Loss of notch1-dependent p21(waf1/cip1) expression influences the notch1 outcome in tumorigenesis. Cell Cycle.

[38] Giuli M.V., Diluvio G., Giuliani E., Franciosa G., Di Magno L., Pignataro M.G., Tottone L., Nicoletti C., Besharat Z.M., Peruzzi G. (2020). Notch3 contributes to t-cell leukemia growth via regulation of the unfolded protein response. Oncogenesis.

[39] Chen J.J., Gao X.T., Yang L., Fu W., Liang L., Li J.C., Hu B., Sun Z.J., Huang S.Y., Zhang Y.Z. (2016). Disruption of notch signaling aggravates irradiation-induced bone marrow injury, which is ameliorated by a soluble dll1 ligand through csf2rb2 upregulation. Sci. Rep..

[40] Antfolk D., Antila C., Kemppainen K., Landor S.K., Sahlgren C. (2019). Decoding the ptm-switchboard of notch. Biochim. Biophys. Acta. Mol. Cell Res..

[41] Nandagopal N., Santat L.A., Elowitz M.B. (2019). Cis-activation in the notch signaling pathway. eLife.

[42] Ong C.T., Cheng H.T., Chang L.W., Ohtsuka T., Kageyama R., Stormo G.D., Kopan R. (2006). Target selectivity of vertebrate notch proteins. Collaboration between discrete domains and csl-binding site architecture determines activation probability. J. Biol. Chem..

[43] Zhou B., Lin W., Long Y., Yang Y., Zhang H., Wu K., Chu Q. (2022). Notch signaling pathway: Architecture, disease, and therapeutics. Signal Transduct. Target. Ther..

[44] Dutta D., Sharma V., Mutsuddi M., Mukherjee A. (2022). Regulation of notch signaling by e3 ubiquitin ligases. FEBS J..

[45] Le Bras S., Loyer N., Le Borgne R. (2011). The multiple facets of ubiquitination in the regulation of notch signaling pathway. Traffic.

[46] Zema S., Pelullo M., Nardozza F., Felli M.P., Screpanti I., Bellavia D. (2020). A dynamic role of mastermind-like 1: A journey through the main (path)ways between development and cancer. Front. Cell Dev. Biol..

[47] Lee H.J., Kim M.Y., Park H.S. (2015). Phosphorylation-dependent regulation of notch1 signaling: The fulcrum of notch1 signaling. BMB Rep..

[48] Rustighi A., Tiberi L., Soldano A., Napoli M., Nuciforo P., Rosato A., Kaplan F., Capobianco A., Pece S., Di Fiore P.P. (2009). The prolyl-isomerase pin1 is a notch1 target that enhances notch1 activation in cancer. Nat. Cell Biol..

[49] Rustighi A., Zannini A., Tiberi L., Sommaggio R., Piazza S., Sorrentino G., Nuzzo S., Tuscano A., Eterno V., Benvenuti F. (2014). Prolyl-isomerase pin1 controls normal and cancer stem cells of the breast. EMBO Mol. Med..

[50] Franciosa G., Diluvio G., Gaudio F.D., Giuli M.V., Palermo R., Grazioli P., Campese A.F., Talora C., Bellavia D., D’Amati G. (2016). Prolyl-isomerase pin1 controls notch3 protein expression and regulates t-all progression. Oncogene.

[51] Palermo R., Checquolo S., Giovenco A., Grazioli P., Kumar V., Campese A.F., Giorgi A., Napolitano M., Canettieri G., Ferrara G. (2012). Acetylation controls notch3 stability and function in t-cell leukemia. Oncogene.

[52] Checquolo S., Palermo R., Cialfi S., Ferrara G., Oliviero C., Talora C., Bellavia D., Giovenco A., Grazioli P., Frati L. (2010). Differential subcellular localization regulates c-cbl e3 ligase activity upon notch3 protein in t-cell leukemia. Oncogene.

[53] Pillidge Z., Bray S.J. (2019). Swi/snf chromatin remodeling controls notch-responsive enhancer accessibility. EMBO Rep..

[54] Giaimo B.D., Oswald F., Borggrefe T. (2017). Dynamic chromatin regulation at notch target genes. Transcription.

[55] Tottone L., Zhdanovskaya N., Carmona Pestaña Á., Zampieri M., Simeoni F., Lazzari S., Ruocco V., Pelullo M., Caiafa P., Felli M.P. (2019). Histone modifications drive aberrant notch3 expression/activity and growth in t-all. Front. Oncol..

[56] Pakkiriswami S., Couto A., Nagarajan U., Georgiou M. (2016). Glycosylated notch and cancer. Front. Oncol..

[57] Pandey A., Niknejad N., Jafar-Nejad H. (2021). Multifaceted regulation of notch signaling by glycosylation. Glycobiology.

[58] Menietti E., Xu X., Ostano P., Joseph J.M., Lefort K., Dotto G.P. (2016). Negative control of csl gene transcription by stress/DNA damage response and p53. Cell Cycle.

[59] Laplane L., Maley C.C. (2024). The evolutionary theory of cancer: Challenges and potential solutions. Nat. Rev. Cancer.

[60] Beumer J., Clevers H. (2024). Hallmarks of stemness in mammalian tissues. Cell Stem Cell.

[61] Shi Q., Xue C., Zeng Y., Yuan X., Chu Q., Jiang S., Wang J., Zhang Y., Zhu D., Li L. (2024). Notch signaling pathway in cancer: From mechanistic insights to targeted therapies. Signal Transduct. Target. Ther..

[62] Wang H., Li J., Du F., Deng H. (2025). Cancer stem cells: Bridging microenvironmental interactions and clinical therapy. Clin. Transl. Med..

[63] Xie Y., Ma S., Tong M. (2022). Metabolic plasticity of cancer stem cells in response to microenvironmental cues. Cancers.

[64] Ceccarelli S., Megiorni F., Bellavia D., Marchese C., Screpanti I., Checquolo S. (2019). Notch3 targeting: A novel weapon against ovarian cancer stem cells. Stem Cells Int..

[65] Giuli M.V., Mancusi A., Giuliani E., Screpanti I., Checquolo S. (2021). Notch signaling in female cancers: A multifaceted node to overcome drug resistance. Cancer Drug Resist..

[66] Gupta P.B., Pastushenko I., Skibinski A., Blanpain C., Kuperwasser C. (2019). Phenotypic plasticity: Driver of cancer initiation, progression, and therapy resistance. Cell Stem Cell.

[67] Matteini F., Thambyrajah R., Montserrat-Vazquez S., Jung S., Ferrer-Perez A., Herrero Molinero P., El Jaramany D., Lozano-Bartolomé J., Mejia-Ramirez E., Gonzalez J. (2026). A notch trans-activation to cis-inhibition switch underlies hematopoietic stem cell aging. Blood.

[68] Thambyrajah R., Maqueda M., Neo W.H., Imbach K., Guillén Y., Grases D., Fadlullah Z., Gambera S., Matteini F., Wang X. (2024). Cis inhibition of notch1 through jagged1 sustains embryonic hematopoietic stem cell fate. Nat. Commun..

[69] Galassi C., Manic G., Esteller M., Galluzzi L., Vitale I. (2025). Epigenetic regulation of cancer stemness. Signal Transduct. Target. Ther..

